# A retrospective chart review of trauma‐related documentation in an Australian substance use treatment service

**DOI:** 10.1111/dar.13575

**Published:** 2022-11-14

**Authors:** Logan R. Harvey, Rachel Hopkins, Melanie Truscott, Christina Marel, Tim Slade, Katherine L. Mills

**Affiliations:** ^1^ Matilda Centre for Research in Mental Health and Substance Use The University of Sydney Sydney Australia; ^2^ Drug Health, Western Sydney Local Health District Sydney Australia

**Keywords:** addiction, complex PTSD, introduction, PTSD, substance use disorder, trauma

## Abstract

**Introduction:**

Trauma exposure among clients of substance use treatment services is almost universal and rates of trauma‐related symptoms are correspondingly high. This study examined one aspect of clinical care—clinical documentation—and sought to systematically assess the documentation of trauma‐related comorbidities and their treatment in a substance use treatment setting.

**Methods:**

A retrospective chart review was conducted on a sample of 300 patient records in a public substance use treatment setting. Rates of documentation of trauma‐related events, symptoms and treatment, along with variables influencing the documentation of these issues, were examined.

**Results:**

Trauma‐related documentation was present in 45.3% of records. There were documented trauma‐related symptoms in 15.3% of records, although treatment activities addressing trauma were only present in 2.3% of records. Being female (odds ratio [OR] 2.58, 95% confidence interval [CI] 1.42, 4.69), having prior mental health treatment (OR 1.82, 95% CI 1.05, 1.12) and having more treatment sessions (OR 1.08, 95% CI = 1.05, 1.12) increased the odds of trauma‐related documentation being present, while being in the first episode of treatment (OR 0.49, 95% CI = 0.28, 0.84) decreased the odds.

**Discussion and Conclusions:**

This study highlights significant under documentation of trauma‐related comorbidities in substance‐use treatment. There is limited evidence of consideration of trauma‐related symptoms or diagnoses, and trauma‐related comorbidities are rarely included in treatment planning activities. The lack of documented trauma‐related information has important clinical and medico‐legal implications for patients, and provides evidence to suggest a lack of integration of treatment for trauma‐related disorders in substance use settings.

**Key Points**

- Trauma‐related comorbidities are inadequately documented during substance use treatment.
- Trauma histories are more regularly documented.
- There is rarely documentation regarding symptoms or diagnoses.
- Trauma‐focused treatment is very rarely planned or documented.
- Patient and treatment characteristics predict documentation regarding trauma.

## 1 INTRODUCTION

Trauma exposure, post‐traumatic stress disorder (PTSD) and substance‐use disorders co‐occur with high frequency [1, 2, 3, 4]. Among people presenting for treatment of substance use issues, trauma exposure is almost universal (80.7–96.0%) and a majority experience multiple trauma types [5, 6, 7]. There are correspondingly high, yet variable, rates of PTSD among people with substance‐use disorders. In a systematic review of Australian studies, Kingston et al. [8] identified estimates of 5–66% for current comorbid diagnosis of PTSD and substance‐use disorders among people entering alcohol and other drug treatment. Accurate clinical records are critical to providing appropriate multidisciplinary care for people with this comorbidity, who often present with a variety of clinical needs that cannot be met by a single clinician [9, 10]. Accurate clinical records are also critical to the provision of person‐centred and trauma‐informed care [11, 12].

Within the substance use treatment field, research has demonstrated that clinicians can underestimate the prevalence of trauma exposure, and fear that trauma inquiry may trigger cravings and relapse [13]. As a result, it is possible that clinicians in these settings may not directly assess trauma histories and related symptoms. Although limited, the available evidence supports this notion, suggesting that trauma inquiry itself is not common [14], and other research indicates documentation of such information is also uncommon. Reynolds et al. [7] compared clinical documentation to information collected from standardised interviews and assessments in patients presenting for substance‐use treatment in the UK. Results indicated that 85% of those who reported trauma exposure during interview had details of their trauma exposure documented in their record; however, there were no documented diagnoses or referrals for treatment. Prior to this, Cole and Sacks [15] undertook a retrospective chart review of 165 patient records in two Australian substance use treatment services. No information on traumatic experiences, trauma‐related symptoms or treatment activities was documented, and only 1% of records referenced a PTSD diagnosis—a rate considerably lower than established prevalence rates in this population [8, 16]. An earlier US study by Dansky et al. [17] also found that although 40% of substance‐use patients of an inpatient psychiatric hospital were diagnosed with PTSD via a structured clinical interview, subsequent review of clinical records identified that only 15% of patients had such a diagnosis documented. In contrast to this, in a sample of female psychiatric inpatients, Xiao et al. [18] found that patients with reported substance use were three times more likely to have trauma histories documented in their records.

In addition to there being limited data on the documentation of trauma, no studies have examined clinician, patient and trauma‐related factors that may influence patterns of documentation in substance use treatment settings. In the broader primary care and mental health literature, several studies have suggested that different professional disciplines document trauma at different rates; however, there is little consistency across these findings [19, 20, 21, 22, 23, 24]. In previous studies, female clinicians were significantly more likely to record trauma‐related information [19, 25, 26], whereas male patients [26] and patients with more severe psychiatric presentations [19, 26, 27, 28] were significantly less likely to have documentation regarding trauma in their clinical records. In regard to the nature of the traumatic events themselves, childhood neglect‐related events have rarely been included in studies and tend to be documented less than abuse‐related events [23, 29]. Even fewer studies have examined the degree to which documented trauma is incorporated into treatment planning. In a New Zealand study of 200 patients receiving treatment at a community mental health centre, only 16.3% of records with an identified trauma history had subsequently incorporated this information into a treatment plan, and only 21.7% were then referred for specific treatment for their trauma‐related issues [19]. The limited evidence of documented referral or treatment in these studies is particularly important to note, given other research indicating that patients for whom a diagnosis is documented tend to have greater symptom severity and receive more treatment, highlighting the clinical importance of accurate assessment and documentation [30].

There are no other identified studies which specifically examine this issue in substance‐use settings, and there are significant methodological shortcomings evident in the available studies, related to data abstraction processes and sampling, limited use of standardised definitions and abstraction forms, and limited examination of issues of reliability of data. Further, the available studies may no longer reflect current practices, particularly given that there have been increasing calls internationally for substance use treatment settings to adopt trauma‐informed practices [11, 31]. Furthermore, there are indications that clinician, patient and trauma‐related factors may influence the likelihood of documentation. As such, the current study aimed to contribute to contemporary understandings of this issue by systematically examining the documentation of trauma and trauma‐related comorbidities in the treatment records of patients attending a public substance use treatment service.

### 1.1 Aims

This study aimed to address the following research questions:

1. What is the prevalence of documented trauma histories in treatment records of people in substance use treatment?
2. What is the prevalence of documented trauma‐related symptoms in treatment records of people in substance use treatment?
3. To what extent are trauma histories or trauma‐related symptoms incorporated into documented treatment plans and activities?
4. Which patient, clinician, event or treatment characteristics are associated with the documentation of trauma‐related information in treatment records?

## 2 METHODS

### 2.1 Design

A retrospective review of medical records was conducted for a random sample of individual patients entering a public substance use treatment service in Australia over a 12‐month period between 1 January 2017 and 31 December 2017. The service provides opiate substitution therapy, counselling and psychology services, drug court and diversion programs, outpatient detoxification and inpatient treatment services for people who use substances.

### 2.2 Procedure

The current study was reviewed by the Westmead Scientific Advisory Quality Assurance Committee and the Secretary of the Western Sydney Local Health District Human Research Ethics Committee and approved as a Quality Assurance Audit Project (QA1906‐07). A total of 1432 intake assessments were completed during the study period. For individuals with multiple intake assessments (*n* = 109), only the earliest completed assessment was retained. One additional assessment was found to be a system‐generated test document and was subsequently excluded. The remaining 1322 patient records were used to generate a random sample of 300 patient records, stratified by sex. The number of records chosen to review was determined by specifying an acceptable level of error around the prevalence estimate of trauma exposure. Utilising an acceptable level of error of ±5% and previous prevalence estimates of 80.7% [5], a minimum sample of 240 records would be required to accurately estimate prevalence [32]. This equates to a relative standard error of 6.2%, far below the recommended 25% typically chosen as the upper limit for reliable prevalence estimates [33]. Hence the current sample will yield prevalence estimates well within the bounds of reliability.

### 2.3 Data abstraction

Patient files were reviewed chronologically, for a period of 12‐months following the initial assessment. This included all available records. Data abstractors were three registered psychologists with postgraduate qualifications in clinical or forensic psychology. All data abstraction was completed using a standardised 52‐item coding form. This form assessed the following.

#### 2.3.1 Patient and treatment process characteristics

Primarily collected as part of routine clinical care based on existing data structures. Patient and treatment characteristics were: sex; age; principal drug of concern; treatment type; treatment status at 12‐months post assessment; the presence of previous mental health treatment; and the presence of previous substance‐use treatment.

#### 2.3.2 Trauma, trauma‐related symptoms and diagnoses

To increase the replicability of the results, these abstraction items were primarily constructed around commonly utilised domains in the published literature. Trauma‐related symptom items were based on the International Classification of Diseases, 11th Revision (ICD‐11) PTSD and complex PTSD diagnostic guidelines [34].

Trauma event categories were derived from the Life Events Checklist for the Diagnostic and Statistical Manual of Mental Disorders, Fifth Edition [35] and the Childhood Trauma Questionnaire [36]. Following a pilot process, it was determined that a large number of incidents of non‐specific domestic and family violence were present but were often unable to be categorised into a specific trauma‐event domain. As such, an additional item was added to document incidents of domestic and family violence which did not provide sufficient information to be categorised as specific events such as ‘*physical assault*’ or ‘*emotional abuse*’. For each traumatic event identified, additional information regarding age at event, number of times documented and in which session type it was documented (e.g., assessment vs. treatment session) was also coded.

#### 2.3.3 Characteristics of the clinician documenting trauma‐related information

Included clinician sex and professional discipline. There were no data available on the staffing levels per discipline or per staff sex for the study period.

### 2.4 Data abstraction pilot process

Initially, abstractors each reviewed the same sample of 10 randomly selected records. Discrepancies were reviewed and discussed until consensus was reached.

### 2.5 Statistical analysis

Intra‐ and inter‐rater reliability were assessed for four broad categories: overall presence of trauma‐related documentation, childhood trauma, adult trauma and trauma symptoms. Intra‐rater reliability was assessed using percent‐agreement between five duplicated charts in each individual abstractor's sample. Inter‐rater reliability was assessed using Krippendorff's Alpha [37], calculated for a random sample of 60 charts (20% of the total sample) which were assessed by all three abstractors.

To assess the predictive value of patient and treatment characteristics for the presence of trauma‐related documentation, a series of bivariate logistic regression analyses were undertaken. Clinician sex and professional discipline could only be collected in instances of documented trauma‐related information and were therefore not included in regression analyses. Odds ratios (OR) with 95% confidence intervals (CI) are reported for each predictor variable. A series of multivariable logistic regression models were then undertaken. All variables found to be statistically significant (*p* < 0.10) in the bivariate models were entered into the initial multivariable model and decisions regarding removal of terms from the model were based on theoretical and statistical grounds. Variables of a *p*‐value >0.10 were systematically removed in a stepped process.

## 3 RESULTS

### 3.1 Reliability analyses

There was 100% agreement across the four categories (overall trauma, childhood trauma, adult trauma and trauma symptoms) for each abstractor, indicating excellent intra‐rater reliability. Inter‐rater reliability was moderate for overall trauma (α = 0.70), childhood trauma (α = 0.76) and adult trauma (α = 0.73), but low for trauma symptoms (α = 0.35).

### 3.2 Sample characteristics

The final sample of records (*n* = 300) included 216 male patients (72.0%) and 84 female patients (28.0%). Patients ranged from 18 to 83 years of age (*M* = 36.25, SD = 10.52). Table 1 provides descriptive information regarding primary substance used and treatment status at 12‐months post‐initial assessment. In total, 52.7% (*n* = 158) of records were documented as the patient's first episode of substance‐use treatment and 57.0% (*n* = 171) had evidence of previous mental health treatment. The most common primary treatment episode type was counselling (*n =* 141, 47.0%), followed by withdrawal management (*n =* 88, 29.3%), pharmacotherapy (*n =* 55, 18.3%) and assessment only (*n =* 16, 5.3%). Thirty‐five percent of records indicated more than one treatment episode type, with secondary episodes including counselling (*n =* 56, 53.3%), pharmacotherapy (*n =* 24, 22.9%), withdrawal management (*n =* 17, 16.2%), assessment only (*n =* 5, 4.8%) and support and case management (*n =* 3, 2.9%). The number of service contacts across the 12‐month review period ranged from 1 to 125 (median 9, interquartile range 12).

**TABLE 1:** Primary substance used and treatment status at 12‐months post‐initial assessment

Variable	*n*	%
Primary substance used		
Opiates	82	27.3
Heroin	54	18.0
Pharmaceutical opioids	28	9.3
Alcohol	77	25.7
Methamphetamine	68	22.7
Cannabis	60	20.0
Benzodiazepines	5	1.7
Cocaine	4	1.3
MDMA	4	1.3
Treatment status at 12 months		
Left without notice	109	36.3
Treatment ongoing	68	22.7
Service completed	57	19.0
Transferred to another service	20	6.7
Imprisoned	19	6.3
Other	16	5.3
Left against advice	11	3.7

### 3.3 What is the prevalence of documented trauma histories in treatment records of people in substance use treatment?

Overall, 45.3% (*n* = 136) of patient records had documented trauma histories, 39.0% (*n =* 117) had documented adult trauma histories and 20.3% (*n* = 61) had documented childhood trauma histories (Table 2). For records with trauma‐related documentation, 32.6% (*n =* 45) reflected both adult and childhood trauma, 52.9% (*n =* 73) reflected only adult trauma and only 14.5% (*n =* 20) reflected solely childhood trauma.

**TABLE 2:** Prevalence of documented traumatic events

	Documented as present	Documented as denied		Not documented		
Trauma type	*n*	%	*n*	%	*n*	%
Any trauma	136	45.3	2	0.7	162	54.0
Adult trauma	117	39.0	1	0.3	182	60.7
Childhood trauma	61	20.3	3	1.0	236	78.7
Domestic violence (unspecified)	55	18.3	1	0.3	244	81.3
Physical assault	29	9.7	0	0	271	90.3
Any other event	25	8.3	0	0	275	91.7
(Childhood) emotional neglect	19	6.3	0	0	281	93.7
(Childhood) emotional abuse	19	6.3	3	1.0	278	92.7
Transportation accident	18	6.0	0	0	282	94.0
(Childhood) sexual abuse	17	5.7	1	0.3	282	94.0
(Childhood) physical abuse	16	5.3	4	1.3	280	93.3
Sexual assault	16	5.3	0	0	284	94.7
Assault with a weapon	11	3.7	0	0	289	96.3
Other serious accident	5	1.7	0	0	295	98.3
Sudden violent death	5	1.7	0	0	295	98.3
Sudden accidental death	5	1.7	0	0	295	98.3
Combat or war exposure	4	1.3	0	0	296	98.7
Life threatening illness or injury	4	1.3	0	0	296	98.7
(Childhood) physical neglect	3	1.0	1	0.3	296	98.7
Severe human suffering	3	1.0	0	0	297	99.0
Fire or explosion	2	0.7	0	0	298	99.3
Serious harm to other	2	0.7	0	0	298	99.3
Captivity	1	0.3	0	0	299	99.7
Natural disaster	0	0	0	0	300	100
Exposure to a toxic substance	0	0	0	0	300	100
Other unwanted sexual event	0	0	0	0	300	100

*Note*: ‘Any trauma’ category does not reflect a cumulative total of the childhood and adulthood categories.

As per Table 2, there were multiple instances of traumatic experiences being documented as ‘*denied*’—indicating that trauma inquiry took place, but that the patient denied having such experiences. This included one instance in which childhood emotional neglect was documented as both present and denied through treatment, suggesting that the patient had responded differently to trauma inquiry at different times.

### 3.4 What is the prevalence of documented trauma‐related symptoms in treatment records of people in substance use treatment?

Prevalence of trauma‐related symptom documentation is presented in Table 3. Eight of 300 records (2.7%) had documentation indicating a prior PTSD diagnosis. There were only two records which documented use of a psychometric screening tool, in both instances the PTSD Checklist for Diagnostic and Statistical Manual of Mental Disorders, Fifth Edition [38].

**TABLE 3:** Prevalence of documented PTSD or Complex PTSD symptoms

	Documented as present		Documented as denied		Not documented	
Symptom	*n*	%	*n*	%	*n*	%
Pre‐existing diagnosis	8	2.7	0	0	292	97.3
Any symptom	46	15.3	2	0.7	252	84.0
Re‐experiencing	22	7.3	0	0	278	92.7
Affect dysregulation	21	7.0	0	0	279	93.0
Negative self‐concept	13	4.3	1	0.3	286	95.3
Avoidance	12	4.0	1	0.3	287	95.7
Difficulties in relationships	11	3.7	0	0	289	96.3
Persistent hyperarousal	6	2.0	0	0	284	94.7
Functional impairment	4	1.3	0	0	296	98.7

Abbreviation: PTSD, post‐traumatic stress disorder.

Psychologists were most commonly the first to document trauma‐related symptoms (65.9%), followed by counsellors (16.5%), doctors (13.2%) and nurses (4.4%). Female clinicians were most commonly the first to document symptoms (57.1%), which were most often first documented in an assessment (55.0%) versus treatment‐focused (45.0%) session.

### 3.5 To what extent are trauma histories or trauma‐related symptoms incorporated into documented treatment plans and activities?

In terms of treatment, there were four records (1.3%) which documented previous trauma‐related treatment. Seven records (2.3%) had evidence of trauma‐related treatment planning in an initial assessment note, while only three records (1.0%) had trauma‐related comorbidities included in a formal treatment plan. Trauma‐related treatment was mentioned in 10 psychology or counselling notes, and in two instances of medical notes. There were nine instances of trauma‐specific interventions, and 18 instances of interventions to reduce trauma‐related symptoms without directly addressing trauma memories.

### 3.6 Which patient, clinician, event or treatment characteristics are associated with the documentation of trauma‐related information in treatment records?

For this analysis, the overall trauma item was recoded such that ‘*Documented as present*’ and ‘*Documented as denied*’ were collapsed into one outcome ‘T*rauma‐related documentation*’. Table 4 displays the results of the bivariate regression analyses. First treatment episode (*χ* ^*2*^[1] = 6.096, *p* = 0.014), multiple treatment types (*χ* ^*2*^[1] = 11.602, *p* = 0.001), previous mental health treatment (*χ* ^*2*^[1] = 11.093, *p* = 0.001), patient sex (*χ* ^*2*^[1] = 17.105, *p* < 0.001), main treatment type (*χ* ^*2*^[3] = 12.360, *p* = 0.006) and number of treatment contacts (*χ* ^*2*^[1] = 23.708, *p* < 0.001) were all found to be significantly related to the presence of trauma‐related documentation. There was little evidence that principal drug of concern (*χ* ^*2*^[4] = 3.109, *p* = 0.540) and patient age (*χ* ^*2*^[1] = 0.258, *p* = 0.612) were associated with the presence of trauma‐related documentation.

**TABLE 4:** Logistic regression predicting the presence of trauma‐related documentation

	Documentation		Bivariate		Final model	
	*n*	%	OR	95% CI	OR	95% CI
Patient age, years	–	–	0.99	0.97, 1.02	–	–
Primary substance						
Alcohol (*n =* 77)	36	46.8	–	–	–	–
Cannabis (*n* = 60)	30	50.0	1.14	0.58, 2.24	–	–
Opiates (*n = 82*)	32	39.0	0.73	0.39, 1.37	–	–
Methamphetamine (*n = 68*)	35	51.5	1.21	0.63, 2.32	–	–
Other (*n = 13*)	5	38.5	0.71	0.21, 2.37	–	–
Multiple treatment types						
No (*n =* 200)	78	39.0	–	–	–	–
Yes (*n =* 100)	60	60.0	2.35**	1.44, 3.83	–	–
Treatment status (+12)						
Completed (*n =* 57)	22	38.6	–	–	–	–
Ongoing (*n =* 68)	43	63.2	2.74**	1.32, 5.66	–	–
Other (*n =* 175)	73	41.7	1.14	0.67, 2.10	–	–
First treatment						
No (*n =* 142)	76	53.5	–	–	–	–
Yes (*n =* 158)	62	39.2	0.56**	0.35, 0.89	0.49**	0.28, 0.84
Main treatment type						
Counselling (*n =* 141)	80	56.7	–	–	–	–
Assessment only (*n =* 16)	5	31.3	0.35*	0.11, 1.05	0.34	0.10, 1.20
Withdrawal management (*n =* 88)	33	37.5	0.46**	0.27, 0.79	0.29***	0.15, 0.56
Pharmacotherapy (*n =* 55)	20	36.4	0.44**	0.23, 0.83	0.18***	0.08, 0.40
Previous mental health treatment						
No (*n =* 129)	45	34.9	–	–	–	–
Yes (*n =* 171)	93	54.4	2.23**	1.39, 3.56	1.82**	1.05, 1.12
Sex						
Male (*n =* 216)	83	38.4	–	–	–	–
Female (*n =* 84)	55	65.5	3.04***	1.80, 5.15	2.58**	1.42, 4.69
# Treatment contacts	–	–	1.07***	1.04, 1.10	1.08***	1.05, 1.12

* *p* < 0.10;

** *p* < 0.05;

*** *p* < 0.001.

The initial multivariable logistic regression model correctly categorised 71.7% of cases and accounted for 33% of the variance in documentation of trauma‐related information (*χ* ^*2*^[10] = 84.402, *p* < 0.001, Nagelkerke *R* ^2^ = 0.33). Being in the first treatment episode (*χ* ^*2*^[1] = 6.369, *p* = 0.012), having received previous mental health treatment (*χ* ^*2*^[1] = 4.098, *p* = 0.043), patient sex (*χ* ^*2*^[1] = 9.242, *p* = 0.002), main treatment type (*χ* ^*2*^[3] = 23.991, *p* ≤ 0.001) and number of treatment contacts (*χ* ^*2*^[1] = 17.588, *p* < 0.001) all contributed significantly to this model. The presence of multiple treatment types (*χ* ^*2*^[1] = 0.150, *p* = 0.698) and treatment status at 12‐months (*χ* ^*2*^[2] = 0.146, *p* = 0.930) did not uniquely contribute to this model and were removed in two steps, starting with the latter. Removal of these variables did not influence any other predictors, or produce any improvement in the model, which correctly classified 71% of cases and accounted for 33% of the variance (*χ* ^*2*^[7] = 84.039, *p* < 0.001, Nagelkerke *R* ^2^ = 0.33). This model was retained as a more parsimonious model.

Within this final model, patient sex was found to be associated with the presence of trauma‐related documentation, such that the odds of having trauma‐related documentation recorded were 2.58 times higher among females than males (95% CI 1.42, 4.69). Being in the first episode of substance‐use treatment halved the odds (OR 0.49, 95% CI 0.28, 0.84) of having trauma‐related documentation present, while the odds of having trauma‐related documentation present were 1.82 times higher among patients who reported a history of previous mental health treatment compared to those who did not (95% CI 1.05, 3.15). In comparison to patients with a primary counselling treatment episode, those with a withdrawal management (OR 0.29, 95% CI 0.15, 0.56) or pharmacotherapy (OR 0.18, 95% CI 0.08, 0.40) primary treatment episode were less likely to have trauma‐related information documented. The odds of trauma‐related material being documented increased by 5% per additional treatment contact (95% CI 1.05, 1.12).

### 3.7 Clinician discipline, clinician sex and session type

For the total sample of identified traumatic experiences across all charts (*n* = 269 events identified in *n* = 137 charts), 46.5% were first documented by a psychologist, 23.8% by a counsellor, 15.6% by a doctor and 14.1% by a nurse. These differences were largest for childhood trauma categories, with psychologists first to document in 63.9% of events, counsellors 15.7%, doctors 14.5% and nurses 6.0%. Female clinicians were most often the first to document trauma (*n* = 181, 67.3%), and trauma‐related information was most often documented in an assessment focused session (*n* = 165, 61.3%) rather than a treatment focused session (*n* = 100, 37.2%), and was documented in both session types rarely (*n* = 4, 1.5%).

## 4 DISCUSSION

The failure of clinicians to document trauma exposure and trauma‐related symptoms or diagnoses has important clinical implications in substance use treatment. This study provides the first systematic examination of trauma‐related documentation in over a decade.

Results indicated that just under half of patient records described traumatic experiences. Childhood trauma experiences were documented less frequently than adult experiences, although where trauma‐related documentation was present, a third of records demonstrated both childhood and adult trauma experiences. This rate is considerably lower than the known prevalence of traumatic events both in the general population and substance using populations [5, 7, 39, 40]. Only eight records documented an established trauma‐related diagnosis, and only 15% documented any trauma‐related symptoms. Of these, there were similar rates of documentation of ICD‐11 PTSD and ICD‐11 complex PTSD symptoms, although the trauma symptom category in this study was found to have low intra‐rater reliability suggesting these symptoms were possibly not clearly documented, or possibly that there could be issues with the definition of these symptoms more broadly.

Previous Australian studies in this population have identified rates of current PTSD between 5% and 66% [8]. The current study identified documented PTSD diagnoses in <3% of patient records, which is similar to previous estimates extracted from treatment records [15]. This pattern is evident in previous studies [7, 17] and suggests that clinicians may be more likely to document the *story* of what happened to a patient, rather than a clinical assessment of subsequent psychopathology.

It is also difficult to know whether the low rates of trauma‐related documentation in this study are reflective of inadequate assessment, or merely reflect hesitancy to document these details. Clinicians may be concerned about permanently documenting a person's trauma history and trauma‐related diagnoses in their medical records due to stigma, and the potential career‐limiting effects of such information [14, 24]. Hesitancy to document may be of particular salience in settings similar to those of the current study, where medical records are accessible to staff across different areas of the broader hospital system, and there are high rates of records being subpoenaed by external agencies, reducing the clinicians' ability to contain this sensitive information. Additionally, in many jurisdictions, in order for patients to access redress or compensation schemes it is necessary that they provide evidence of previous disclosure or treatment seeking for trauma‐related issues [41]. As such, if this information is not clearly documented, patients may be disadvantaged when making relevant applications later. Further, the standardised clinical documents in the current service did not include specific fields for trauma or related symptoms, only a non‐specific option to document reported pre‐existing mental health diagnoses. There are also very limited options available for treatment of these comorbidities in the current setting—meaning that clinicians may not be inclined to assess or consider these comorbidities, if they are not able to refer a patient for specialist trauma‐focussed treatment.

Rates of documented trauma‐related treatment activities in the current study were also low, as was the inclusion of trauma‐related comorbidities in treatment plans; a finding consistent with previous studies in psychiatric samples [19]. The lack of inclusion of trauma‐related comorbidities in treatment planning is concerning, as previous studies have highlighted a link between trauma‐related documentation and the likelihood of subsequent treatment activities [30].

Female patients were found to be more than twice as likely to have trauma‐related information documented. Previous studies in similar populations have not demonstrated significant sex differences in overall rates of trauma exposure [5, 42], although this current finding is consistent with other chart review studies in psychiatric samples which found female patients were more likely to have trauma‐related documentation in their records [26].

While unable to be included in regression analyses, the general pattern of allied health staff more frequently documenting trauma‐related information is also consistent with previous literature [20, 21]. This finding suggests that differences in clinical approach and training may exist among multi‐disciplinary teams and highlights that there is an inconsistent approach to documenting trauma‐related comorbidities.

In the current study only two records had evidence of psychometric screening measures being utilised. Previous research has identified that even when services have trauma‐specific items included in standardised assessment forms, these are often inadequately or inconsistently used [22, 25, 43, 44]. It is likely that the use of psychometric measures would provide a useful and standardised approach to assessing trauma‐related comorbidities and informing treatment planning and interventions. Broader implementation of trauma‐informed care principles in this setting (e.g., [45]) and access to training options may assist clinicians to more consistently consider these comorbidities. Previous studies have demonstrated that targeted training can assist in both overcoming clinician barriers to trauma inquiry, but also increase frequency of inquiry [14, 46]. This is particularly important given that less than two‐thirds of clinicians in this area report having received any trauma training, meaning that many clinicians may be hesitant to explore this due to a lack of knowledge and skills [47].

The findings of the current study need to be considered in light of some limitations. It is possible that the actual clinical activities being undertaken are more substantial than those reflected in clinical records. Furthermore, the current information was collected in a single service and may not generalise to other treatment services. The low reliability of trauma‐symptom data is a further limitation, but this appears to be an artefact of the documentation of these symptoms as opposed to the methods of the current study. This study focused on symptoms of PTSD and complex PTSD; however, it is important to acknowledge that the impacts of trauma exposure can extend beyond these symptoms (e.g., [48]) and future research investigating this issue in other populations is recommended. These limitations highlight the need for future research to consider how best to capture the characteristics of treatment activities in real world settings to further examine how this issue is addressed in different treatment services.

Trauma‐related comorbidities are prevalent and influential and present unique challenges for substance use treatment settings. Although these comorbidities are well studied, and evidence‐based treatment exists, the accurate assessment and documentation of these comorbidities is crucial for treatment activities. The current study highlights significant inadequacies and inconsistencies in documentation practices even within a single service. As such, the implementation of trauma‐informed models of care and specific practices to assess and document trauma‐related comorbidities is imperative for clinical services.

## FUNDING INFORMATION

Logan R. Harvey is supported by a Matilda Centre PhD Scholarship. Katherine L. Mills was supported by an Australian Government National Health and Medical Research Council Senior Research Fellowship. Christina Marel was supported by an Australian Government National Health and Medical Research Council Translating Research into Practice Fellowship.

## CONFLICT OF INTEREST

None.
